# Focal Therapy for Prostate Cancer: A Treatment Option Reserved for the Ideal Candidate?

**DOI:** 10.1590/S1677-5538.IBJU.2026.0222

**Published:** 2026-06-20

**Authors:** Daniele Cignoli, João Paulo Barbosa de Oliveira, Andrea Rodriguez-Serrano, David Lopez, Armando Stabile, Andrea Salonia, Alberto Briganti, Francesco Montorsi, Giovanni La Croce, Michele Catellani, Paolo Barzaghi, Marco Roscigno, Luigi Filippo Da Pozzo, Yenny Arroyo Rojas, Julio César González Brambila, Camille Lanz, Eric Barret, Petr Macek, Xavier Cathelineau, Lara Rodriguez-Sanchez

**Affiliations:** 1 Institut Montsouris Department of Urology Paris France Department of Urology, Institut Montsouris, Paris, France; 2 ASST Papa Giovanni XXIII Department of Urology Bergamo Italy Department of Urology, ASST Papa Giovanni XXIII, Bergamo, Italy; 3 Vita-Salute San Raffaele University IRCCS San Raffaele Scientific Institute Urological Research Institute Milan Italy Unit of Urology, Division of Experimental Oncology, Urological Research Institute, IRCCS San Raffaele Scientific Institute, Vita-Salute San Raffaele University, Milan, Italy; 4 Hospital Joya Guadalajara Guadalajara Mexico Hospital Joya Guadalajara. Endourología y Cirugía Robótica. Guadalajara, Mexico; 5 Université Paris Cité Paris France Université Paris Cité, Paris, France

**Keywords:** Prostatic Neoplasms, Cryotherapy, High-Intensity Focused Ultrasound Ablation

## Abstract

**Objectives:**

To evaluate outcomes of FT (HIFU or cryotherapy) in prostate cancer (PCa) patients who do not meet the ideal focal therapy candidate profile, assessing whether expanding selection criteria beyond traditional parameters is safe.

**Materials and Methods:**

A retrospective analysis was performed on 282 patients treated with FT for ISUP 1 and 2 PCa at a single European center (2009–2020). Patients were categorized into three groups: G1 (ideal candidates: single lesion ≤12mm, favorable biopsy features), G2 (ISUP 1 with minor deviations), and G3 (ISUP 2 with multiple lesions, larger tumor size, or positive cores outside the target zone). A secondary exploratory analysis compared ISUP 2 patients fully meeting criteria versus those with one deviating feature. Multivariable regression with propensity score matching was applied.

**Results:**

Overall recurrence occurred in 187 patients (66%): 77% in G1, 60% in G2, and 77% in G3. No significant differences were observed between G1 and G3 regarding infield recurrence-free survival, clinically significant infield recurrence-free survival, or outfield recurrence-free survival (all p>0.05). In the secondary analysis, lesion size ≥12mm was the only feature associated with worse infield recurrence-free survival (p=0.04) and clinically significant infield recurrence-free survival (p=0.001). Multivariate Cox regression confirmed lesion size as an independent predictor (HR: 1.21; 95% CI: 1.05–1.40; p=0.01).

**Conclusions:**

Expanding FT selection criteria to include selected ISUP 2 patients with features outside traditional parameters appears safe. These preliminary findings highlight the need for multicenter studies to validate these results.

## INTRODUCTION

Prostate cancer [PCa] is a prevalent disease characterized by significant clinical, morphological, and molecular heterogeneity (1). Consequently, treatment approaches also vary (2). Radical treatments for localized PCa, such as radical prostatectomy and radiotherapy, can adversely affect quality of life (3). Focal therapy (FT) has emerged as a promising alternative (4), demonstrating safety and potential benefits regarding genitourinary function and oncological outcomes. However, more extensive follow-up studies are required to move from hypothesis and preliminary findings to robust evidence (5, 6).

Advances in imaging and targeted biopsy have improved the ability to differentiate intermediate- and high-grade cancers from indolent ones. If accurately targeted, according to consensus meetings (5, 7-10), FT could optimize treatment for localized, small-volume cancers in intermediate-risk patients and selected high-risk individuals. Conversely, indolent cancer areas, such as Grade Group (GG) 1 lesions, can be monitored via active surveillance, particularly in patients with GG2 disease where only GG1 areas are targeted for active treatment. One of the latest consensus on the field suggests that in cases of clinically localized cancers with favorable single lesions, FT could be appropriate for men with ISUP 2 PCa, PSA <15 ng/mL, a single visible lesion ≤ 12 mm, with a maximum of one positive ISUP 1 core outside the target lesion (8–10).

Despite potential, recent literature displays significant variability in selection criteria, treatment methods, relapse definitions, follow-up protocols, and outcome reporting. While some studies advocate FT for higher-grade diseases without compromising metastasis or cancer-specific survival (5), optimal PSA levels and lesion size specifications remain debated. Consequently, current EAU and AUA guidelines advise caution, recommending FT within clinical trials or prospective registries (11, 12).

Identifying ideal candidates for FT thus remains a pivotal topic of discussion. We hypothesized that selected patients not strictly fulfilling the traditional FT inclusion criteria could still achieve oncological outcomes similar to those of ideal candidates. This study therefore aimed to evaluate selection criteria and the outcomes of patients not meeting the ideal FT profile, to determine whether expanding the criteria is both safe and effective.

## MATERIAL AND METHODS

### Study population and design

This retrospective observational study relied on a prospectively maintained institutional database from a single European tertiary referral center, covering patients treated for low- to intermediate-risk PCa with FT between 2009 and 2020. According to local institutional policies for retrospective anonymized database analyses, formal ethics committee approval and informed consent were not required. Inclusion criteria included patients over 18, treatment-naïve, with mpMRI and subsequent biopsy confirming low- to intermediate-risk localized PCa (following the risk stratification of the "Guidelines on Prostate Cancer" proposed by the EAU and associated entities at the time of data analysis and patient follow-up, in 2023 (2)) having primary FT (Cryotherapy, Ablatherm^®^ HIFU, Focal One^®^ HIFU). Considering these criteria, out of 356 patients treated with FT, 282 were analyzed, and categorized into three groups. Both patients with MRI-visible and MRI non-visible lesions were included, as long as clinically localized PCa had been confirmed at targeted and/or systematic biopsy. The three groups were: Group 1 = perfect candidates (30 patients) following all the selection criteria recommended by the FT community, especially the International Delphi Consensus Project (2, 5, 8–10) (ISUP 2, PSA<15ng/mL, single visible lesions ≤12 mm at pre-treatment MRI, max 1 positive core ISUP 1 outside target lesion at pre-treatment biopsy); Group 2 (low-risk ISUP 1 patients, 181 patients); Group 3 (intermediate-risk not included in Group 1, 71 patients). Furthermore, a secondary exploratory analysis only considering ISUP 2 was conducted, comparing Group 1 to patients who were not ideal candidates for only one single criterion. These were divided into 3 groups: Sub-group 2 = with a single pre-treatment MRI lesion>12mm (5 patients); Sub-group 3 = with more than 1 lesion at pre-treatment MRI (9 patients); Sub-group 4 = with more than 1 positive core outside target lesion at pre-treatment biopsy (28 patients). Study design is summarized in Supplementary Table-1.

### Follow-up protocol

Because of the long inclusion period and the lack of dedicated guideline-based follow-up protocols for focal therapy during the years considered, follow-up was not fully standardized and changed over time according to institutional practice and to emerging evidence. In general, however, patients were followed with PSA every 3–6 months in the first 2 years and at least once a year afterwards. mpMRI was usually performed within the first year after treatment, and then repeated based on clinical suspicion. Follow-up biopsies were either protocol-driven (within 12–18 months after FT) or for-cause, triggered by a PSA rise, suspicious mpMRI findings or clinical concern. Retreatment or salvage therapy was offered in case of clinically significant recurrence, multifocal disease progression, radiological progression, or based on patient preference (Supplementary Figures 1, 2 and 3).

### Treatment protocol

Treatment was planned based on mpMRI findings together with targeted and systematic biopsy results. FT was delivered either as hemiablation or as quadrant ablation, depending on lesion location, size and laterality. Hemiablation was usually chosen for larger unilateral lesions, and quadrant ablation for smaller focal lesions. Whenever technically possible, a safety margin around the index lesion was included.

### Variables and outcomes definition

Data collected included patient demographics, baseline PCa features, and details on FT type and extent. Follow-up data included PSA values, PSA nadir, % of PSA reduction (defined as the ratio between PSA nadir and pre-treatment PSA), MRI and biopsies results. Recurrence was defined as any positive biopsy after FT. Infield recurrence referred to recurrence within the treated area, whereas outfield recurrence referred to recurrence outside the treated area. Clinically significant recurrence was defined as ISUP ≥2 disease. Local progression was defined as radiological and/or histological progression within the treated field.

The primary outcome was recurrence-free survival (time between FT and any positive biopsy during follow-up), comparing Group 1 to Group 2 and Group 3. Recurrence-free survival was further analyzed in pre-specified subgroup analyses according to: (i) location of recurrence (infield vs outfield), and (ii) ISUP grade at recurrence (ISUP 1 only vs ISUP ≥2, the latter defined as clinically significant recurrence). Secondary outcomes included progression-free survival (time between FT and local or distant disease progression, death, or end of follow-up), second treatment-free survival (time between FT and salvage/second treatment), and overall survival (time between FT and death or end of follow-up).

An exploratory secondary analysis compared Group 1 to Sub-group 2 (single pre-treatment MRI lesion >12 mm), Sub-group 3 (more than 1 lesion at pre-treatment MRI), and Sub-group 4 (more than 1 positive core outside the target lesion at pre-treatment biopsy), in terms of recurrence-free survival, infield recurrence-free survival, clinically significant infield recurrence-free survival, outfield recurrence-free survival, second treatment-free survival, progression-free survival, and overall survival.

## Statistical analysis

Statistical methodologies followed validated guidelines (13). Comparisons involved chi-square tests, Fisher's exact tests, and Kruskal-Wallis tests as applicable. Multivariable regression analysis combined with propensity score matching minimized confounding among groups and Kaplan-Meier (KM) survival analysis was conducted. Covariates included: pre-treatment PSA (as a continuous variable), PSAd (as a continuous variable), MRI lesion dimension (as a continuous variable), presence of a single visible lesion, and presence of positive biopsies outside the target lesion (more than 1 positive core ISUP 1 outside target lesion). Furthermore, multivariate Cox regression model, between intermediate-risk patients, was fitted to predict how pre-treatment PSA, PSAd, MRI lesion dimension, presence of a single visible lesion and presence of positive biopsies outside target lesion might influence survival analysis. The R software environment for statistical computing and graphics (version 3.4.3) was used for all the statistical analyses. All tests were two-sided, and the significance level was set at p<0.05.

## RESULTS

### Baseline and Treatment Features of the Study Cohort

Baseline and treatment features of the study cohort, stratified by group, are summarized in Table-1. No significant differences were noted in pre-treatment PSA, prostate volume, and PSAd between groups. Patients in group 1 exhibited worse clinical staging compared to group 2, with a higher cT2 rate (33% vs. 17%, p=0.003). No differences were found between groups 1 and 3 (p=0.7). Group 1 was characterized by having only one visible lesion at MRI, whereas 6% (n=11) of group 2 and 32% (n=23) of group 3 had more than one lesion (p=0.001). Group 1 patients had a higher occurrence of PI-RADS 4 and 5 lesions compared to group 2 (77% vs. 29%, p<0.001). Lesion dimensions at pre-treatment MRI were similar between groups 1 and 2 (10 mm, IQR: 8-12, p=0.3) but lower than group 3 (12 mm, IQR: 10-14, p<0.001). Group 3 patients had a greater median number of positive cores [3 (IQR: 2-4) vs. 2 (IQR: 1-3), p=0.002] and a longer length [14mm (7-21) vs. 7mm (IQR:3-10), p=0.03]. No differences in Gleason 4 percentages were noted between groups 1 and 3 (p=0.6). Cancer adjacent to the target zone was found in 27% (n=8) of group 1 and 21% (n=38) of group 2 (all ISUP 1), while systematic adjacent biopsy was positive in 75% (n=53) of group 3, with a rate of ISUP 2 at 65% (n=46, p<0.001). Systematic positive biopsies away from the target zone were 0% in group 1, 3% (n=5) in group 2, and 24% (n=17) in group 3, with an ISUP 2 rate of 14% (n=10) in group 3 (p=0.01). Focal One^®^ HIFU was performed in most patients in groups 1 (60%, n=18) and 3 (56%, n=40), while the majority in group 2 underwent Ablatherm^®^ HIFU (45%, n=81, p<0.001). Cryotherapy rates were 20% (n=6) in group 1, 39% (n=71) in group 2, and 24% (n=17) in group 3. Quadrant ablation rates were lower in group 1 (43%, n=13) compared to groups 2 (72%, n=130) and 3 (69%, n=49). Group 1 predominantly underwent hemiablation (57%, n=17) compared to group 2 (28%, n=51) and group 3 (31%, n=22, p=0.01 and p=0.02, respectively).

**Table 1:** Baseline, treatment, and follow-up features of 282 patients treated with focal therapy for prostate cancer, stratified by group.

Variables		Group 1 (30)	Group 2 (181)	Group 3 (71)	p G1/G2 (p G1/G3)
**Baseline characteristics**					
Age (years), Median (IQR)		68 (62-71)	67 (62-72)	69 (62-74)	0.6 (0.9)
pre-treatment PSA (ng/mL), Median (IQR)		6.1 (4.6-8.9)	6.8 (5.7-9.5)	5.7 (5.7-5.7)	0.1 (0.05)
Prostate volume (mL), Median (IQR)		42 (35-48)	40 (34-50)	40 (34-49)	0.7 (0.7)
PSAd (ng/mL^2^), Median (IQR)		0.2 (0.1-0.2)	0.2 (0.1-0.2)	0.2 (0.1-0.3)	0.2 (0.07)
**cT, n (%)**					**0.003 (0.7)**
	1	20 (67)	151 (83)	53 (75)	
	2	10 (33)	30 (17)	18 (26)	
**pre-treatment MRI single lesion, n (%)**					**0.3 (0.001)**
	No	0 (0)	11 (6)	23 (32)	
	Yes	30 (100)	170 (94)	48 (68)	
**Pre-treatment MRI PIRADS, n (%)**					**<0.001 (0.2)**
	2	1 (3)	82 (45)	0 (0)	
	3	6 (20)	46 (25)	15 (21)	
	4	20 (67)	42 (23)	40 (56)	
	5	3 (10)	11 (6)	16 (23)	
MRI lesion dimension (mm), Median (IQR)		10 (8-12)	10 (8-12)	12 (10-14)	0.3 (<0.001)
**Pre-treatment biopsy ISUP, n (%)**					<**0.001 (0.2)**
	1	0 (0)	181 (100)	0 (0)	
	2	30 (100)	0 (0)	71 (100)	
**Pre-treatment biopsy ISUP systematic adjacent, n (%)**					**0.6 (<0.001)**
	0	22 (73)	143 (79)	18 (25)	
	1	8 (27)	38 (21)	7 (10)	
	2	0 (0)	0 (0)	46 (65)	
**Pre-treatment biopsy ISUP systematic non-adjacent, n (%)**					**0.8 (0.01)**
	0	30 (100)	176 (97)	54 (76)	
	1	0 (0)	5 (3)	7 (10)	
	2	0 (0)	0 (0)	10 (14)	
**Treatment**					
	**Treatment modality, n (%)**				**<0.001 (0.9)**
	Cryotherapy	6 (20)	71 (39)	17 (24)	
	Ablatherm^®^ HIFU	6 (20)	81 (45)	14 (20)	
	Focal One^®^ HIFU	18 (60)	29 (16)	40 (56)	
**Year of treatment, n (%)**					**<0.001 (0.8)**
	2009-2011	2 (6)	54 (30)	2 (2)	
	2012-2014	8 (27)	79 (44)	22 (32)	
	≥2015	20 (66)	48 (27)	47 (67)	
**Treatment extension, n (%)**					**0.01 (0.02)**
	Quadrant ablation	13 (43)	130 (72)	49 (69)	
	Hemiablation	17 (57)	51 (28)	22 (31)	
**Follow-up and outcomes**					
	PSA nadir (ng/mL), Median (IQR)	2.2 (1.4-3.8)	2.7 (1.4-4.4)	2.2 (1.3-3.8)	0.5 (0.9)
	%PSA reduction, Median (IQR)	63 (37-77)	60 (39-78)	64 (51-82)	0.9 (0.4)
**Recurrence, n (%)**					**0.1 (0.9)**
	No	7 (23)	72 (40)	16 (23)	
	Yes	23 (77)	109 (60)	55 (77)	
**Infield recurrence, n (%)**					**0.3 (0.7)**
	No	11 (37)	88 (49)	22 (31)	
	Yes	19 (63)	93 (51)	49 (69)	
**Clinically significant infield recurrence, n (%)**					**0.3 (0.1)**
	No	19 (63)	134 (74)	32 (45)	
	Yes	11 (37)	47 (26)	39 (55)	
**Outfield recurrence, n (%)**					**0.9 (0.8)**
	No	22 (73)	139 (77)	49 (69)	
	Yes	8 (27)	42 (23)	22 (31)	
**Recurrence ISUP, n (%)**					**0.5 (0.6)**
	Negative	7 (23)	73 (40)	16 (23)	
	1	8 (27)	48 (27)	10 (14)	
	2	10 (33)	41 (23)	28 (39)	
	3	4 (13)	15 (8)	11 (16)	
	4	1 (3)	3 (2)	6 (9)	
	5	0 (0)	1 (1)	0 (0)	
**Pathological upgrading, n (%)**					**0.1 (0.6)**
	No	25 (83)	121 (67)	54 (76)	
	Yes	5 (17)	60 (33)	17 (24)	
**Second treatment, n (%)**					**0.6 (0.8)**
	No	12 (40)	85 (47)	25 (35)	
	Yes	18 (60)	96 (53)	46 (65)	
**Second treatment type, n (%)**					**0.8 (0.3)**
	None	12 (40)	80 (44)	24 (34)	
	HIFU	3 (10)	15 (8)	6 (9)	
	Cryotherapy	0 (0)	8 (4)	2 (3)	
	sRP	6 (20)	15 (8)	5 (7)	
	EBRT	3 (10)	25 (14)	18 (25)	
	Brachytherapy	2 (7)	12 (7)	7 (10)	
	ADT	3 (10)	13 (7)	3 (4)	
	sRP + ADT	0 (0)	1 (1)	0 (0)	
	EBRT + sRP	0 (0)	3 (2)	0 (0)	
	EBRT + ADT	1 (3)	7 (4)	6 (9)	
	Brachytherapy + ADT	0 (0)	1 (1)	0 (0)	
	sRP + ADT + EBRT	0 (0)	1 (1)	0 (0)	
**Progression, n (%)**					**0.8 (1)**
	No	27 (90)	169 (93)	65 (92)	
	Yes	3 (10)	12 (7)	6 (9)	
**Progression site, n (%)**					**0.1 (0.1)**
	Bone	1 (25)	3 (23)	5 (71)	
	Liver	1 (25)	0 (0)	0 (0)	
	Pelvic	1 (25)	6 (46)	1 (14)	
	Retroperitoneal	1 (25)	4 (31)	1 (14)	
**Death, n (%)**					**0.9 (0.9)**
	No	30 (100)	178 (98)	69 (97)	
	Yes	0 (0)	3 (2)	2 (3)	

PSA = prostate-specific antigen; PSAd = PSA density; cT = clinical stage; MRI = magnetic resonance imaging; PIRADS = Prostate Imaging-Reporting and Data System; ISUP = International Society of Urological Pathology grade; HIFU = high-intensity focused ultrasound; sRP = salvage radical prostatectomy; EBRT = external beam radiation therapy; ADT = androgen deprivation therapy. All deaths observed in the cohort were due to causes other than prostate cancer; no cancer-specific deaths were recorded. Additional pre-treatment biopsy variables (number and percentage of positive cores, positive length in mm and percentage of positive mm, and percentage of Gleason pattern 4) used in the baseline profile only are reported in Supplementary Table-2

### Follow-up and survival analysis

Follow-up features of the study cohort after FT for PCa, stratified by group, are summarized in Table-2. Median follow-up for the whole cohort was 75 months (IQR 54–105), calculated from FT to recurrence, death or last follow-up.

No statistical differences were found in terms of PSA nadir and %PSA reduction between groups (Table-2, Supplementary Figure-4). Median PSA nadir was 2.2 ng/mL (IQR 1.4–3.8) in G1, 2.7 ng/mL (IQR 1.4–4.4) in G2, and 2.2 ng/mL (IQR 1.3–3.8) in G3, with a median %PSA reduction of 63% (IQR 37–77), 60% (IQR 39–78), and 64% (IQR 51–82), respectively (all p>0.05). No statistical difference was detected for local recurrence rate in any prostate site (infield vs outfield) nor ISUP grade (ISUP 1 vs ISUP ≥2). Local recurrence rates ranged from 60% to 77% (G1: 77% [23/30]; G2: 60% [109/181]; G3: 77% [55/71]); infield recurrence rate ranged from 51% to 69%; clinically significant infield recurrence rate ranged from 26% to 55%; clinically significant outfield recurrence ranged from 23% to 31%. Pathological upgrading at recurrence was experienced by 17% (n=5) in G1, 33% (n=60) in G2, and 24% (n=17) in G3 compared to baseline ISUP grade.

Recurrence-free survival was higher for patients in group 2 compared to those in group 1. KM estimates indicated a median time to any recurrence of 68 months (95% confidence interval [CI]: 52-90) for group 2 vs. 18 months (95%CI: 13-56) for group 1 (p<0.001). Infield recurrence-free survival was also significantly higher for group 2 compared to group 1 (p=0.006), as well as infield clinically significant free survival (p=0.02). No differences were observed in terms of outfield recurrence-free survival between the two groups (p=0.2). Additionally, no specific differences were noted in recurrence rates, infield recurrence-free survival, infield clinically significant free survival, and outfield free survival between groups 1 and 3, all p>0.05, Figure-1.

**Figure 1:**
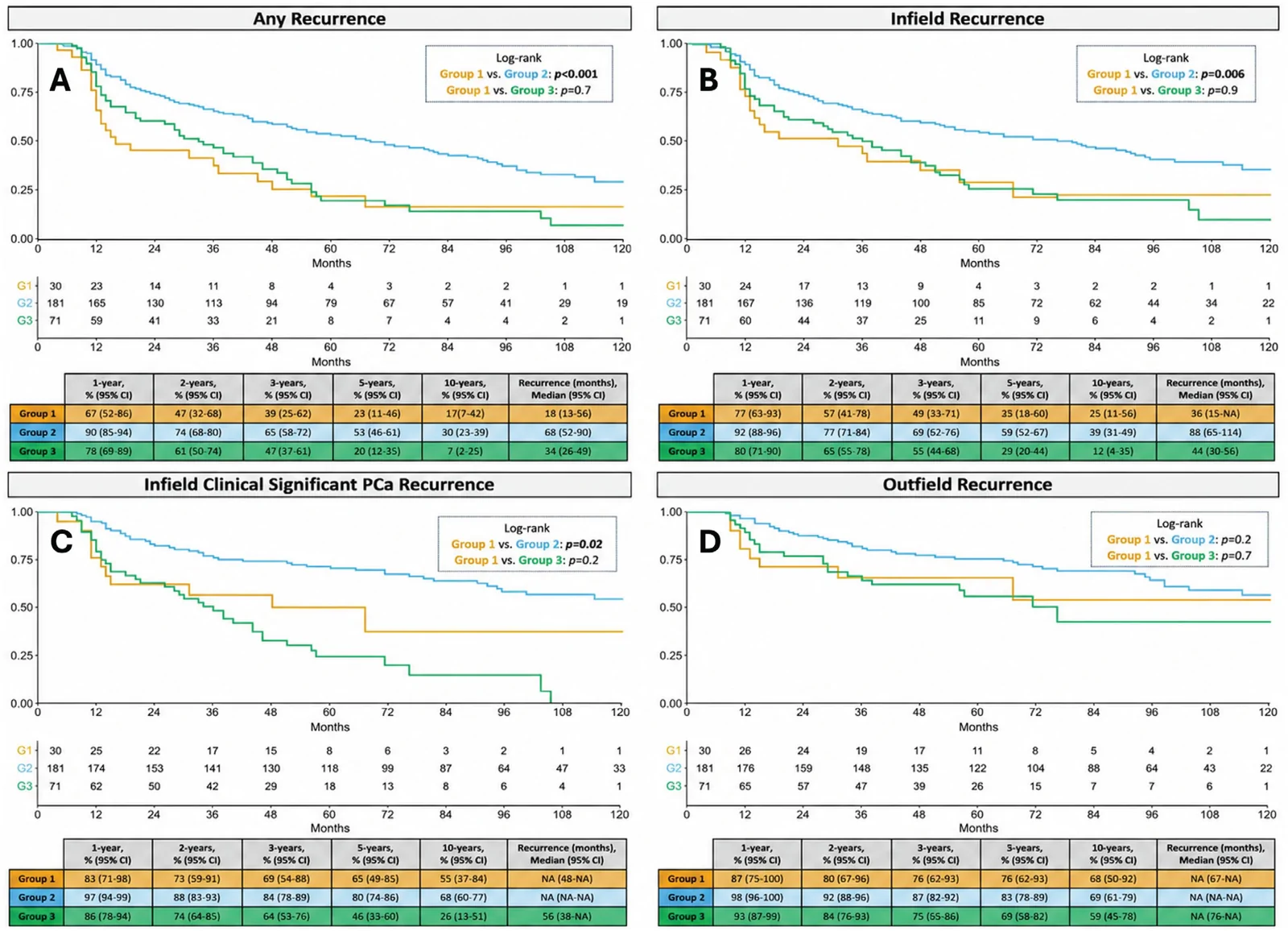
Kaplan–Meier curves assessing any recurrence-free survival, infield recurrence-free survival, infield clinically significant PCa recurrence-free survival and outfield recurrence-free survival (Time 0 was time of first focal treatment for prostate cancer) of 282 patients treated with FT for PCa divided into 3 groups based on baseline PCa features.

Panel A) any recurrence-free survival; Panel B) infield recurrence-free survival; Panel C) infield clinically significant PCa recurrence-free survival; Panel D) outfield recurrence-free survival.

Group 1 = perfect candidates (30 patients) following all the inclusion criteria recommended by the FT community (2,5,8–10) (ISUP 2, PSA<15ng/mL, single visible lesions ≤12 mm at pre-treatment MRI, max 1 positive core ISUP 1 outside target lesion at pre-treatment biopsy);

Group 2 = low-risk PCa (ISUP 1) (181 patients);

Group 3 = intermediate-risk PCa not included in Group 1 (71 patients).

Of the 187 patients presenting with recurrence after FT, 86% (n=160) required additional treatment, while 14% (n=27) underwent active surveillance due to low-risk disease. Active treatments listed in Table-2 included repeat FT using the same or different energy sources; salvage RP (sRP); salvage radiotherapy (sRT); androgen deprivation therapy (ADT); or multimodal treatment. Second treatment-free survival was higher for patients in group 2 compared to group 1, with KM estimates showing a median time to second treatment of 87 months (95% CI: 70-101) for group 2 compared to 50 months (95% CI: 35-not reached) for group 1 (p=0.04). No differences were found between groups 1 and 3 (p=0.9), Supplementary Figure-5. 21 patients (7%) experienced oncologic progression, and 5 patients (2%) died (no cancer-specific deaths were observed), although no statistically significant differences were found regarding progression-free survival and overall survival between the three groups, Supplementary Figure-6.

### Secondary exploratory analysis

In the secondary exploratory analysis, no differences emerged between the perfect candidates (Group 1) and the almost perfect candidates with more than one lesion at pre-treatment MRI (Sub-group 3), as well as those with more than one positive core outside the target lesion at pre-treatment biopsy (Sub-group 4), in terms of any recurrence-free survival, infield recurrence-free survival, infield clinically significant free survival, and outfield free survival (Figure-2). KM estimates revealed a median time to any recurrence of 18 months (95% CI: 13-56) for Group 1, 13 months (95%CI: 9-not reached) for Sub-group 2, 56 months (95%CI: 28-not reached) for Sub-group 3, and 28 months (95%CI: 14-71) for Sub-group 4 (Figure-2). A statistically significant difference was observed only between Group 1 and Sub-group 2 in terms of infield recurrence-free survival [36 months (95%CI: 15-not reached) vs. 13 months (95%CI: 9-not reached), p=0.04] and infield clinically significant free survival [not reached (95%CI: 48-not reached) vs. 13 months (95%CI: 9-not reached), p=0.001] (Figure-2).

**Figure 2:**
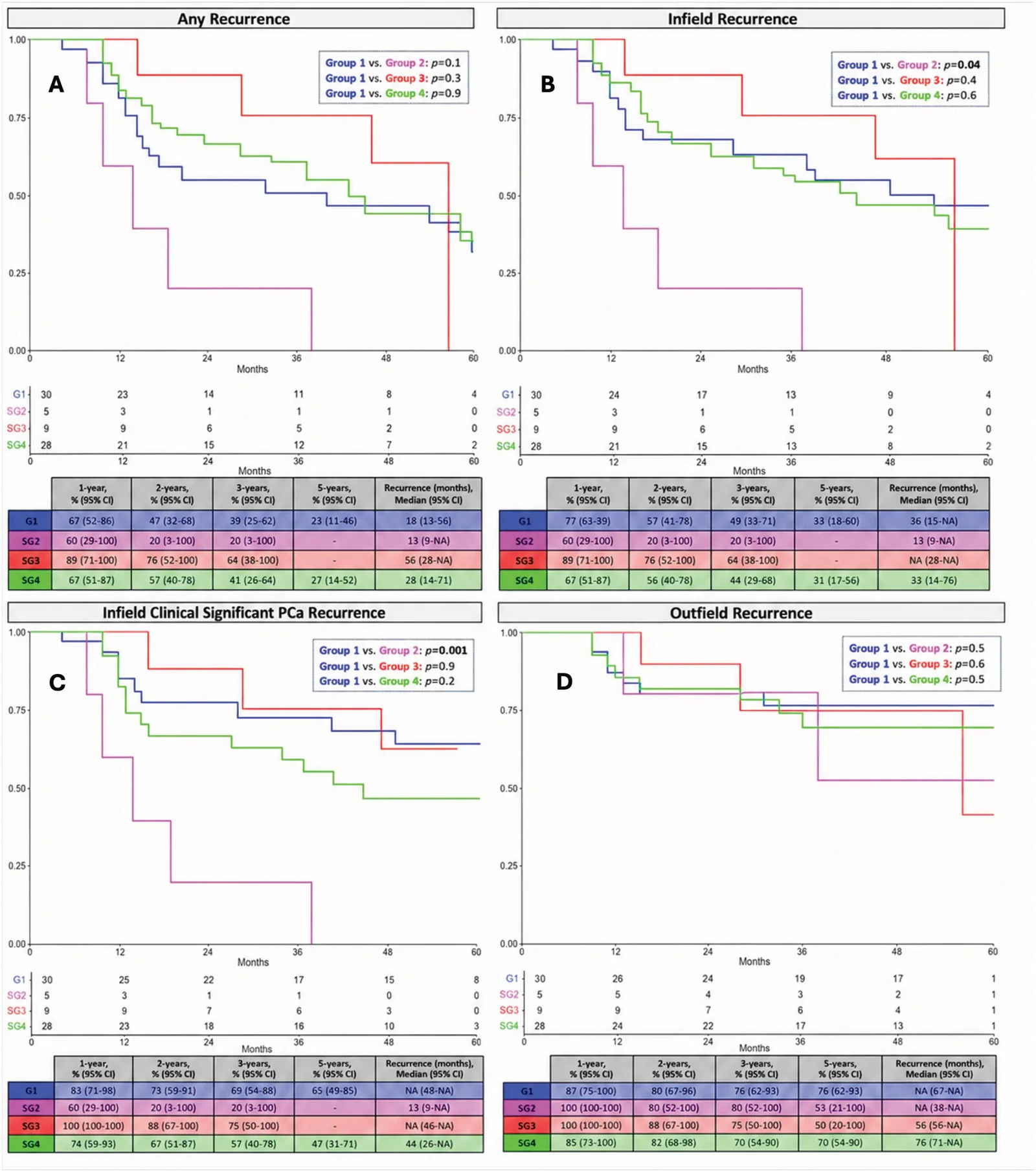
Kaplan–Meier curves assessing any recurrence-free survival, infield recurrence-free survival, infield clinically significant PCa recurrence-free survival and outfield recurrence-free survival (Time 0 was time of first focal treatment for prostate cancer) of the secondary exploratory analysis population (only considering intermediate-risk PCa).

Panel A) any recurrence-free survival; Panel B) infield recurrence-free survival; Panel C) infield clinically significant PCa recurrence-free survival; Panel D: outfield recurrence-free survival. In all panels, "Sub-Group 2", "Sub-Group 3", and "Sub-Group 4" replace the previous "Group 2", "Group 3", and "Group 4" labels.

Group 1 = perfect candidates (30 patients) following all the inclusion criteria recommended by the FT community (2,5,8–10) (ISUP 2, PSA<15ng/mL, single visible lesions ≤12 mm at pre-treatment MRI, max 1 positive core ISUP 1 outside target lesion at pre-treatment biopsy);

Sub-group 2 = with a single pre-treatment MRI lesion > 12mm (5 patients);

Sub-group 3 = with more than 1 lesion at pre-treatment MRI (9 patients);

Sub-group 4 = with more than 1 positive core outside target lesion at pre-treatment biopsy (28 patients).

Multivariate Cox regression analyses indicated that lesion dimension (based on the maximum lesion diameter) independently influenced infield clinically significant recurrence-free survival (hazard ratio [HR]: 1.21; 95% CI: 1.05-1.40; p=0.01) (Table-3). The relationship between lesion size and the predicted probability of clinically significant recurrence appeared to be highly linear based on the estimates derived from the Cox model, with other covariates fixed at their mean values. Notably, the predicted probability exceeded 70% for lesions longer than 12 mm (Figure-3). Furthermore, the presence of more than one positive core outside the target lesion at pre-treatment biopsy independently influenced outfield recurrence-free survival (HR: 3.22; 95%CI: 1.18-8.76; p=0.02) (Table-3).

**Figure 3:**
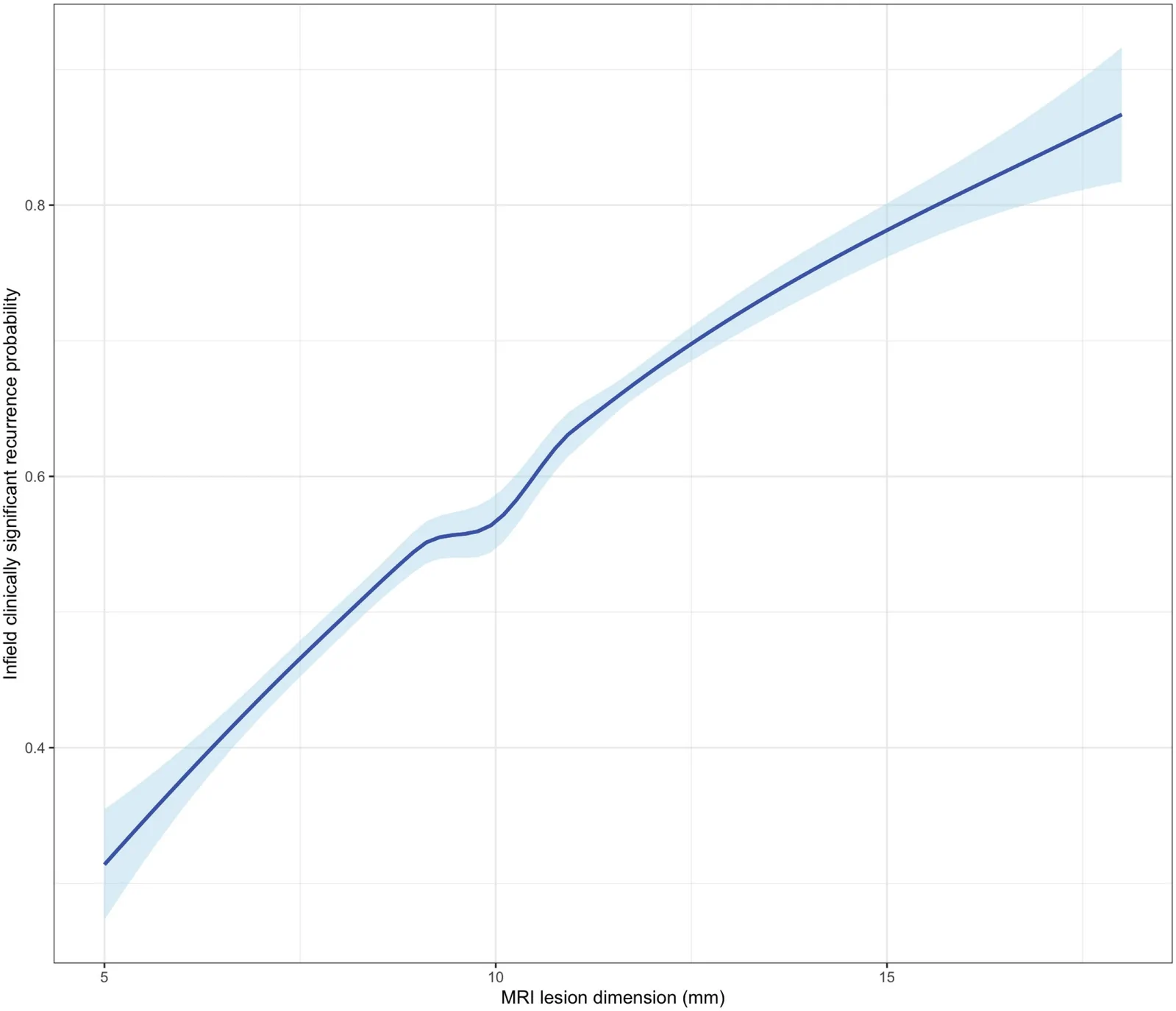
Probability of infield clinically significant PCa recurrence according to mpMRI lesion dimension in 72 patients treated with FT for intermediate-risk PCa.

The curve represents the predicted probability of infield clinically significant prostate cancer recurrence (ISUP ≥2) as a function of maximum mpMRI lesion diameter, derived from the multivariate Cox regression model with all other covariates fixed at their mean values. The shaded area represents the 95% confidence interval. The relationship was highly linear, with predicted probability exceeding 70% for lesions >12 mm.

## DISCUSSION

The selection criteria for successful focal therapy (FT) vary significantly across the literature (5, 8–10, 14, 15). Study protocols differ regarding PSA thresholds, local staging (based on digital rectal examination or MRI), ISUP grades, the number of visible lesions on MRI, and volume limits of the index lesion. This study investigates the feasibility and safety of expanding FT selection criteria for localized PCa. The results offer insights into patient selection, highlighting key determinants that influence treatment outcomes.

Our findings indicate that FT can be safely administered to patients who do not fit the traditional "ideal" candidate profile, typically characterized by strict criteria regarding PSA levels, lesion size, and biopsy results. Notably, no significant differences were found in long-term outcomes such as progression-free survival and overall survival between ideal candidates (Group 1) and intermediate-risk patients who did not meet these criteria (Group 3). This suggests that broader selection criteria could enable more patients to benefit from FT without compromising their prognoses. Additionally, no differences were found between the perfect candidates (Group 1) and the almost perfect candidates with more than 1 lesion at pre-treatment MRI (Sub-group 3) and the almost perfect candidates with more than 1 positive core outside target lesion at pre-treatment biopsy (Sub-group 4) in terms of any recurrence free survival, infield recurrence free survival, infield clinically significant free survival and outfield free survival. However, patients with MRI-detected lesions larger than 12 mm (Sub-group 2) experienced significantly higher rates of infield recurrence compared to ideal candidates, suggesting that lesion size may be an important factor influencing FT success. Of note, Sub-group 2 included only 5 patients, so this result needs further validation in larger series.

The notion that FT could be effective for higher-risk patients aligns with earlier studies. For instance, Guillaumier et al. found that FT could control disease in intermediate- and high-risk patients with careful patient selection (16). Reddy et al. (17) analyzed data from 1,379 patients in the HIFU Evaluation and Assessment of Treatment (HEAT) registry, which included ISUP GG3 PCa. While differences in failure-free survival between ISUP GG2 and GG3 were significant, the study reported 7-year metastasis-free and PCa-specific survival rates exceeding 99%. Moreover, Scheltema et al. (18) noted a median 5-year outcome for primary FT in localized PCa, including a subset of ISUP GG3 patients, finding no significant differences in failure-free survival per ISUP grade, suggesting FT could extend to high-risk PCa with correct patient selection.

As noted, larger pre-treatment MRI lesion dimensions correlate with a higher risk of clinically significant infield recurrence, emphasizing the critical importance of accurate imaging and targeted biopsy for identifying suitable FT candidates. Previous studies indicate that lesion characteristics, particularly size and location, are vital in predicting FT success, with larger lesions resulting in higher recurrence rates post-FT (19–21). Additionally, positive biopsies outside the target area at pre-treatment biopsy predict an increased risk of outfield recurrence. It is acknowledged that multiparametric MRI (mpMRI) can underestimate tumor boundaries by up to 10 mm (22), advocating for at least a 10 mm treatment margin to ensure complete ablation (23). Studies, such as the one by Aslim et al. (24), emphasize how intralesional heterogeneity affects mpMRI performance, and advanced imaging techniques could enhance outcomes. Integrating MRI-histology co-registration for defining three-dimensional treatment margins may also improve precision (23), and the concepts of "umbra" and "penumbra" underline the need for comprehensive perilesional sampling (25). Future advancements in prostate mpMRI aim to improve resolution and target lesion definition, potentially enhancing FT outcomes.

Of note, among emerging imaging techniques, PSMA-PET has attracted considerable interest for its potential both in guiding initial treatment planning and in evaluating response after FT. Although the current body of evidence is preliminary and not yet definitive, the potential applications of PSMA-PET in this setting are encouraging (26). Additional studies are essential to verify these possibilities and to broaden the integration of this promising modality into routine clinical practice.

Meticulous patient selection and precise targeting remain crucial objectives for FT's effectiveness (27, 28). Our research supports that expanding FT criteria to include more GG2 and selected GG3 diseases can be both safe and beneficial, provided lesion-specific factors are carefully considered.

The recurrence rates seen in our series were somewhat higher than in some published cohorts. This is likely due to a longer follow-up, a more intensive post-treatment biopsy protocol used in the early FT era, and the inclusion of historical cases treated before improvements in mpMRI, targeting and focal therapy technology. The gap between the median time to recurrence and the median time to second treatment in Group 1 (18 vs. 50 months) can also be explained by the fact that some patients with low-risk recurrence were kept on active surveillance before salvage treatment. Our results are in line with other recent IBJU contributions on this topic, including the meta-analysis by Andrade et al. on ablative versus radical treatments (29), the nomogram by Kaneko et al. for predicting csPCa in patients with negative MRI (30), the work of Porcaro et al. on disease progression in intermediate-risk patients (31), the review by Ezequiel et al. on barriers to focal therapy in South America (32), and the series by Mate et al. on focal cryotherapy stratified by Gleason score (33), which also supports the idea that ISUP grade alone is not enough to select FT candidates.

Strengths of this study include a comprehensive analysis of a large, prospectively maintained institutional database. The use of multivariable regression analysis combined with propensity score matching adds statistical rigor to the findings. However, the study also has limitations. The observational nature of the study could introduce biases that are not entirely mitigable by statistical adjustments. Moreover, the study is based on a single high-volume center, which may limit the generalizability of the results to other healthcare settings with different patient populations and resources. An important limitation of the study is the lack of a standardized protocol for follow-up may contribute to variability in treatment outcomes and complicate comparisons across patient populations. This underscores the necessity for future multicenter studies to standardize protocols, thereby enhancing the consistency and reliability of outcomes achieved with FT for PCa. Another limitation of this study is the inclusion of a substantial proportion of ISUP 1 patients, which poses challenges in interpreting treatment outcomes; while we advocate active surveillance as the primary management strategy for these patients, this study reflects also a period when focal therapy was more commonly applied to low-risk disease. To address this issue, we treated ISUP 1 patients as a separate group in the analysis. Furthermore, the small sample sizes of certain subgroups definitively limit the statistical power and precision of these findings. As these are secondary exploratory analysis, they cannot be used to inform clinical practice but should serve as a basis for further research. Additionally, changes in the PI-RADS version (PIRADS v1 vs. PIRADS v2) during the study period could have influenced the observed differences between groups, potentially affecting the consistency and comparability of the results. Furthermore, historical series dating back to 2009, along with evolving practices in MRI utilization, cancer detection methods (such as the types of biopsies), and advancements in focal treatment technologies, have likely influenced some outcomes, including recurrence rates and the need for additional treatment.

## CONCLUSIONS

Expanding FT selection criteria in localized PCa, including GG2 and selected GG3 disease, appears safe and acceptable, allowing more patients to benefit from FT. However, the importance of lesion size as a critical factor influencing treatment success highlights the need for careful patient selection and tailored follow-up strategies. Future studies are needed to validate these findings in larger, multicenter cohorts to enhance generalizability and prospective clinical trials are warranted to further investigate the long-term outcomes of expanded FT criteria and to establish standardized protocols for monitoring and managing post-FT recurrences.

## Data Availability

Trial data will be made available on reasonable request to the corresponding author.
