# Supplementary material for: Focal Therapy for Prostate Cancer: A Treatment Option Reserved for the Ideal Candidate?
Source: Int Braz J Urol. 2026 Jun 20;52(5):e20260222. doi: 10.1590/S1677-5538.IBJU.2026.0222 (PMC13682755; doi:10.1590/S1677-5538.IBJU.2026.0222)
Supplement: Supplementary file 1 [file 1677-6119-ibju-52-5-e20260222-Suppl01.pdf]

APPENDIX 1

Supplementary material

Supplementary Table-1

	ISUP = 2	PSA < 15 ng/mL	Single visible lesion at mpMRI	Lesion ≤ 12 mm	Max 1 positive core ISUP 1 outside target lesion
**Group 1 (30 pts)**	Yes	Yes	Yes	Yes	Yes
**Sub-group 2 (5 pts)**	Yes	Yes	Yes	No	Yes
**Sub-group 3 (9 pts)**	Yes	Yes	No	Yes	Yes
**Sub-group 4 (28 pts)**	**Yes**	**Yes**	**Yes**	**Yes**	**No**

Supplementary Figure 1 - Percentage of patients who performed biopsy at different time points during follow-up after focal treatments for prostate cancer.

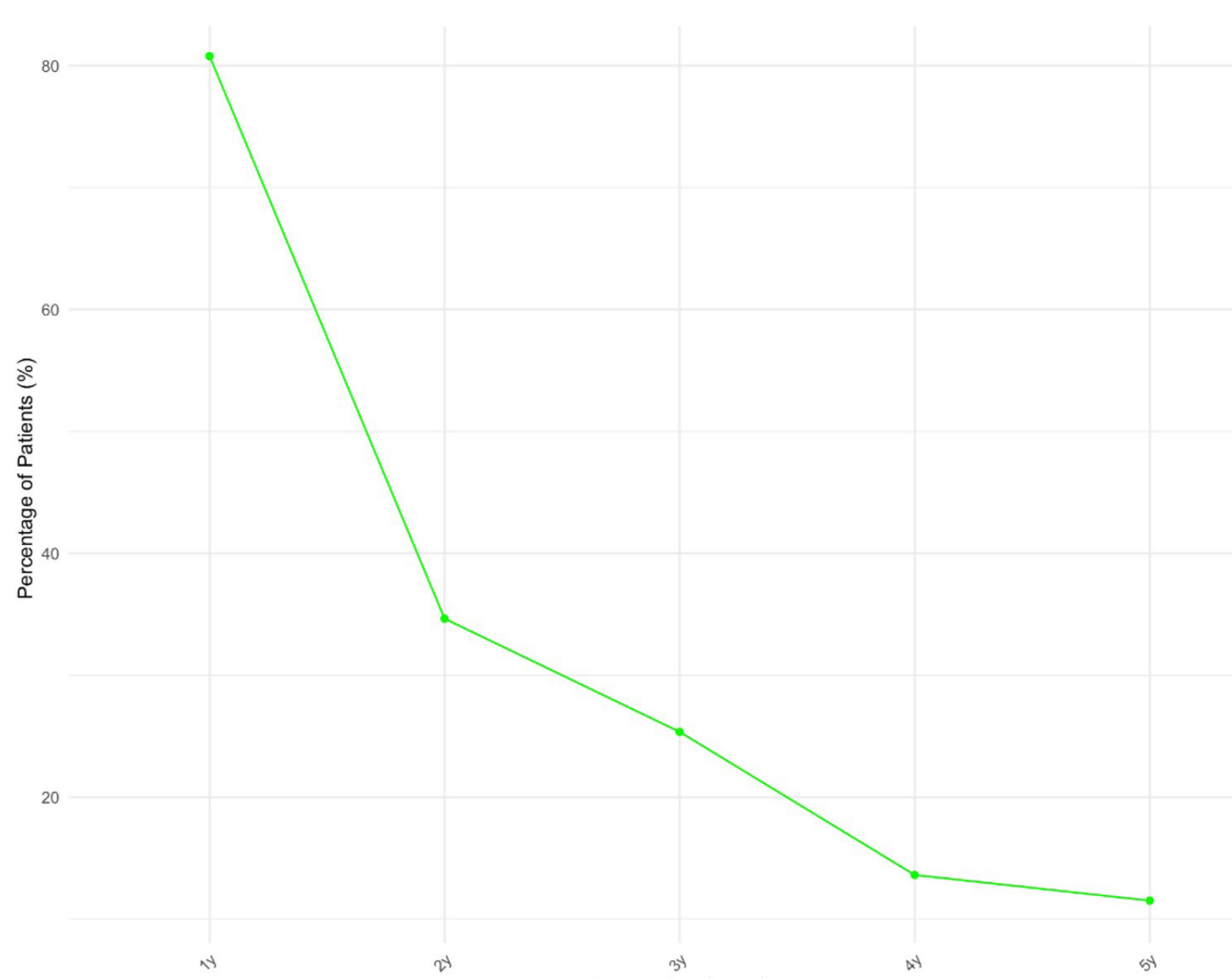

Percentages calculated based on patients at risk (without recurrence) at each time point

Supplementary Figure 2 - Percentage of patients who performed multiparametric MRI at different time points during follow-up after focal treatments for prostate cancer.

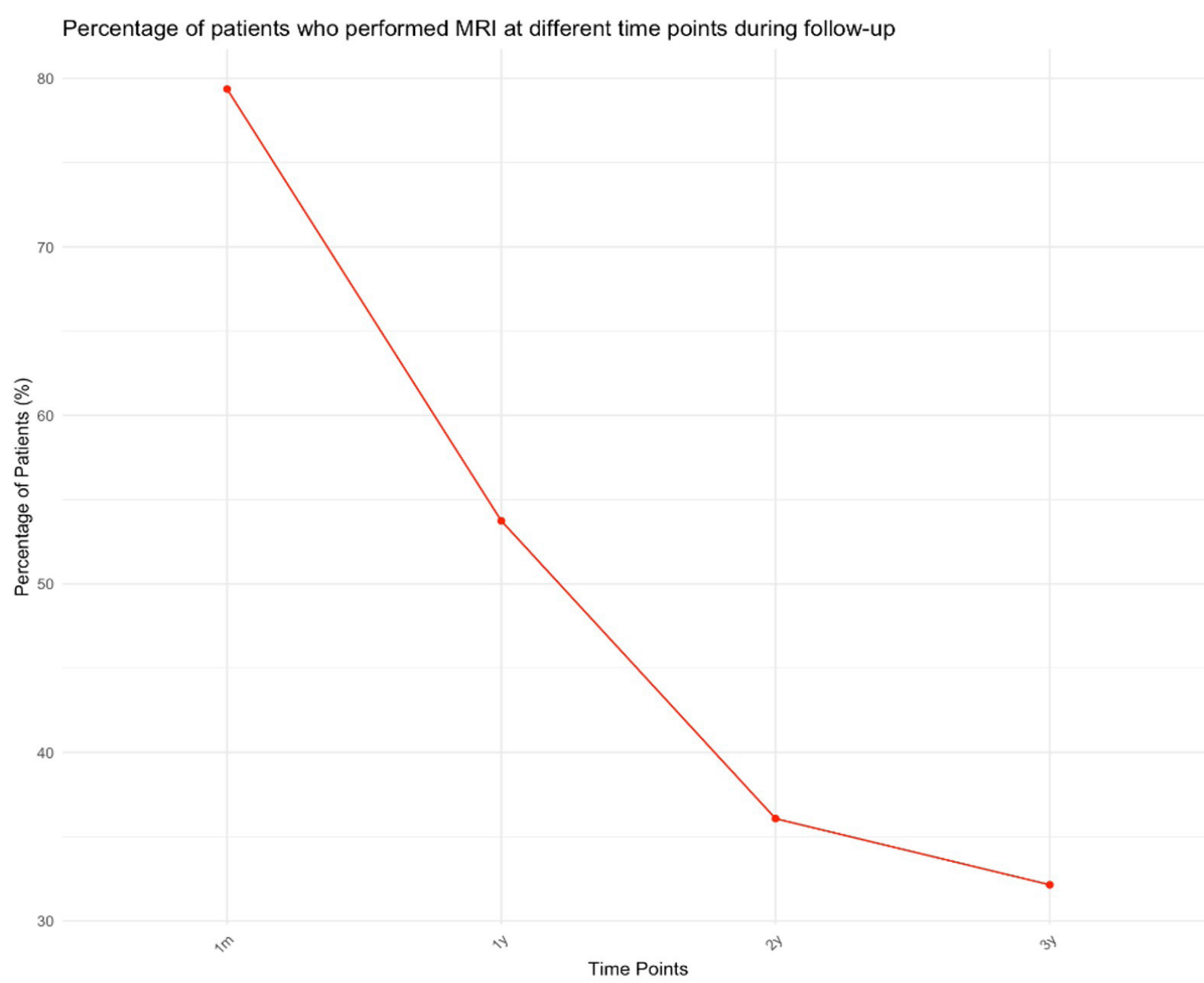

Percentages calculated based on patients at risk (without recurrence) at each time point.

Supplementary Figure 3 - Percentage of patients who performed PSA at different time points during follow-up after focal treatments for prostate cancer.

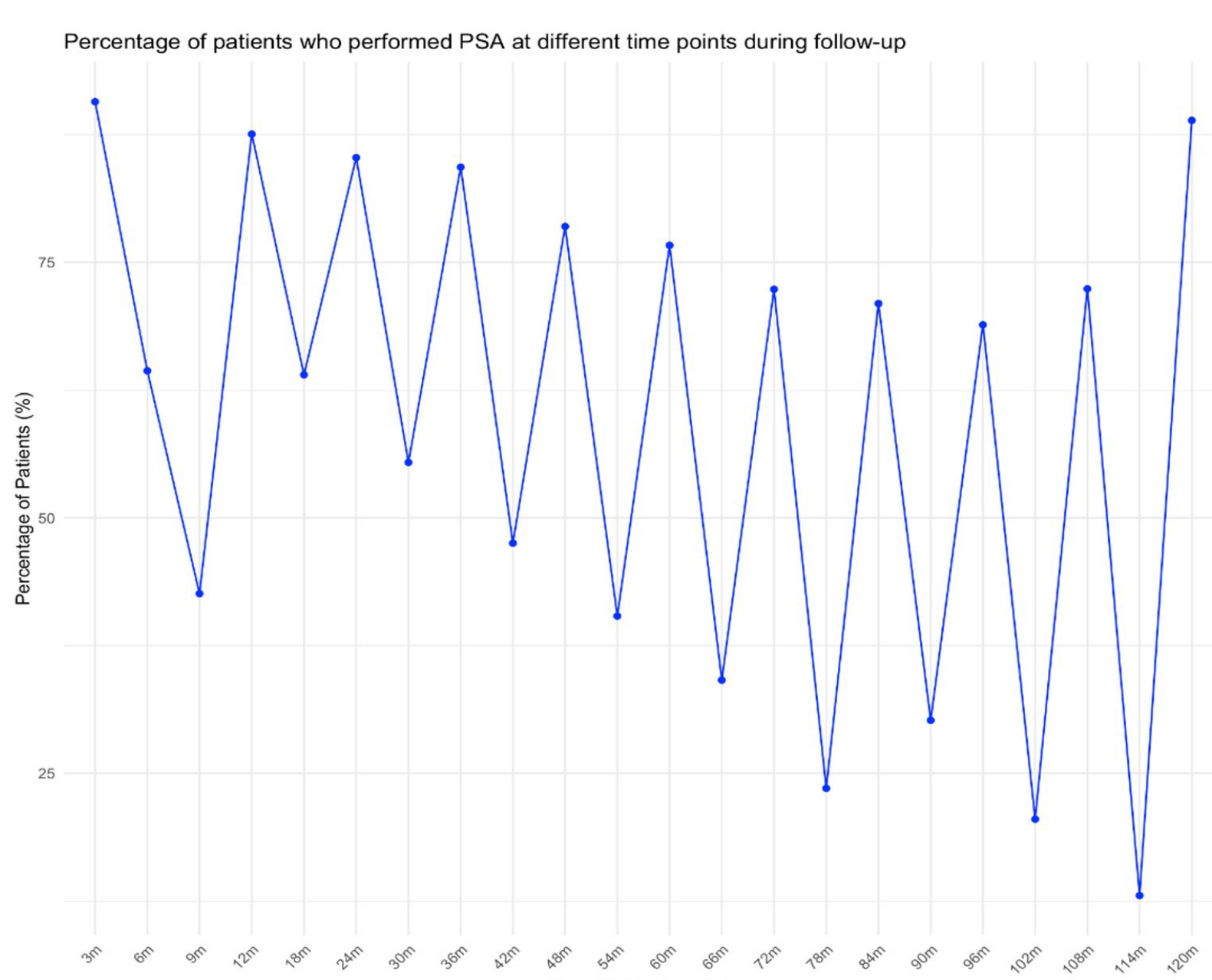

Percentages calculated based on patients at risk (without recurrence) at each time point.

Supplementary Figure 4 - Violin plot of PSA nadir and % PSA reduction after focal treatment for prostate cancer in 282 patients, stratified by group.

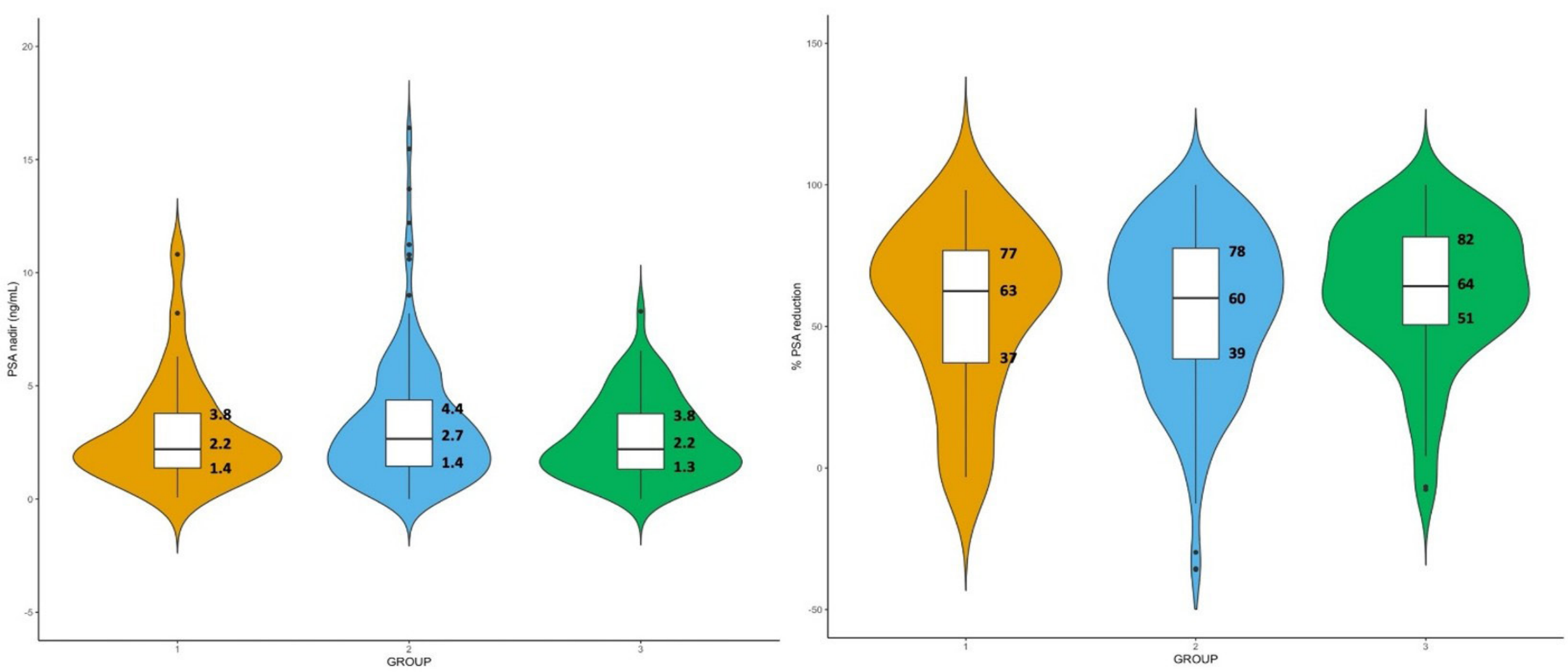

Supplementary Figure 5 - Kaplan-Meier curves assessing second treatment-free survival (Time 0 was time of first focal treatment for prostate cancer).

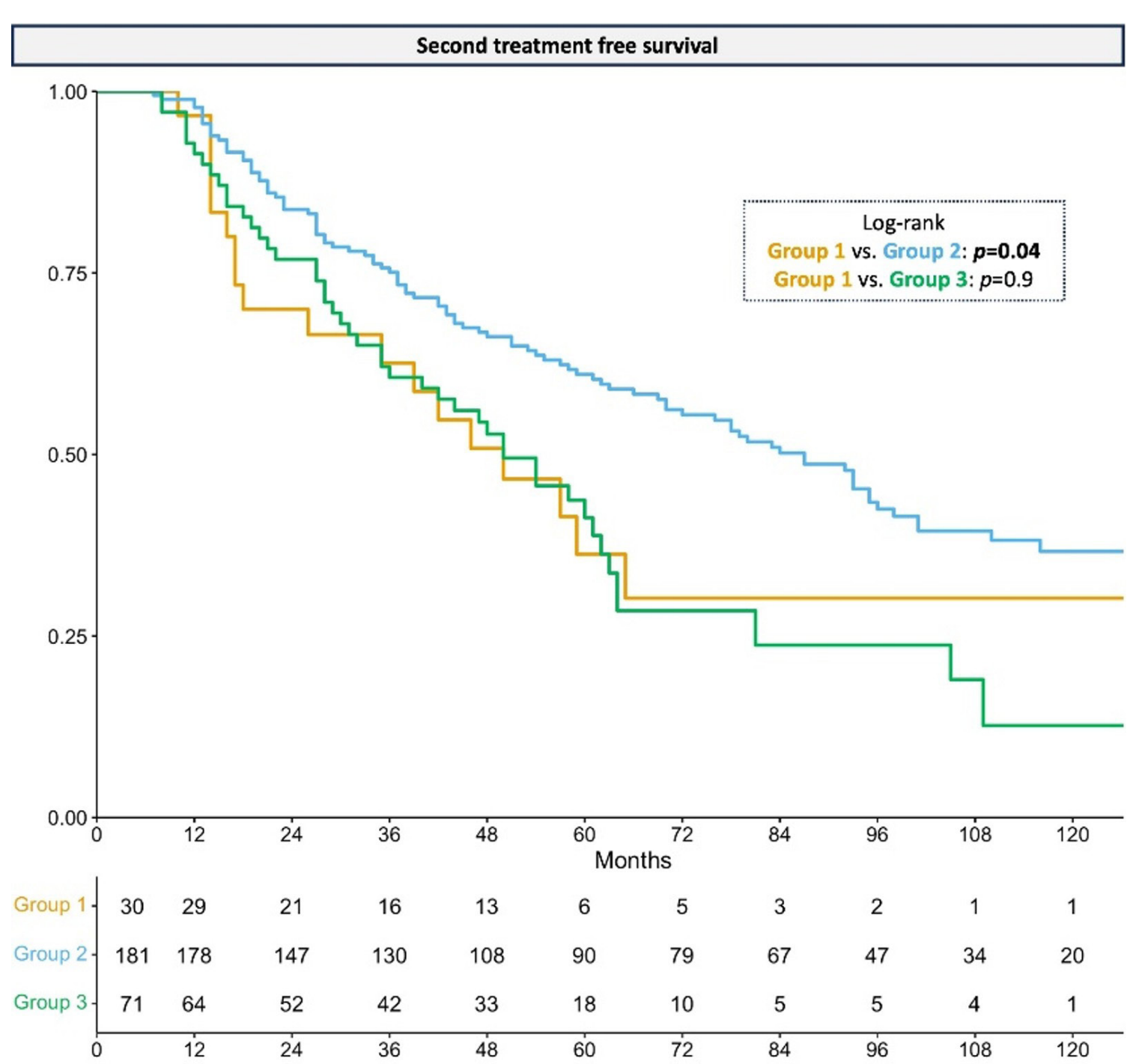

	1-year, % (95% CI)	2-years, % (95% CI)	3-years, % (95% CI)	5-years, % (95% CI)	10-years, % (95% CI)	Recurrence (months), Median (95% CI)
Group 1	83.3 (71.0-97.8)	66.5 (51.5-85.8)	58.7 (43.1-79.9)	36.3 (21.2-61.9)	30.2 (15.9-57.5)	50 (35-NA)
Group 2	97.8 (95.7-100)	83.2 (77.8-88.8)	75.1 (69.0-81.8)	60.4 (53.4-68.2)	36.7 (29.1-46.2)	87 (70-101)
Group 3	91.4 (85.1-98.2)	73.9 (64.2-85.0)	60.6 (50.1-73.4)	41.3 (30.5-55.9)	12.7 (4.3-37.2)	50 (40-63)

Supplementary Figure 6 - Kaplan-Meier curves assessing progression-free survival and all-cause mortality (Time 0 was time of first focal treatment for prostate cancer).

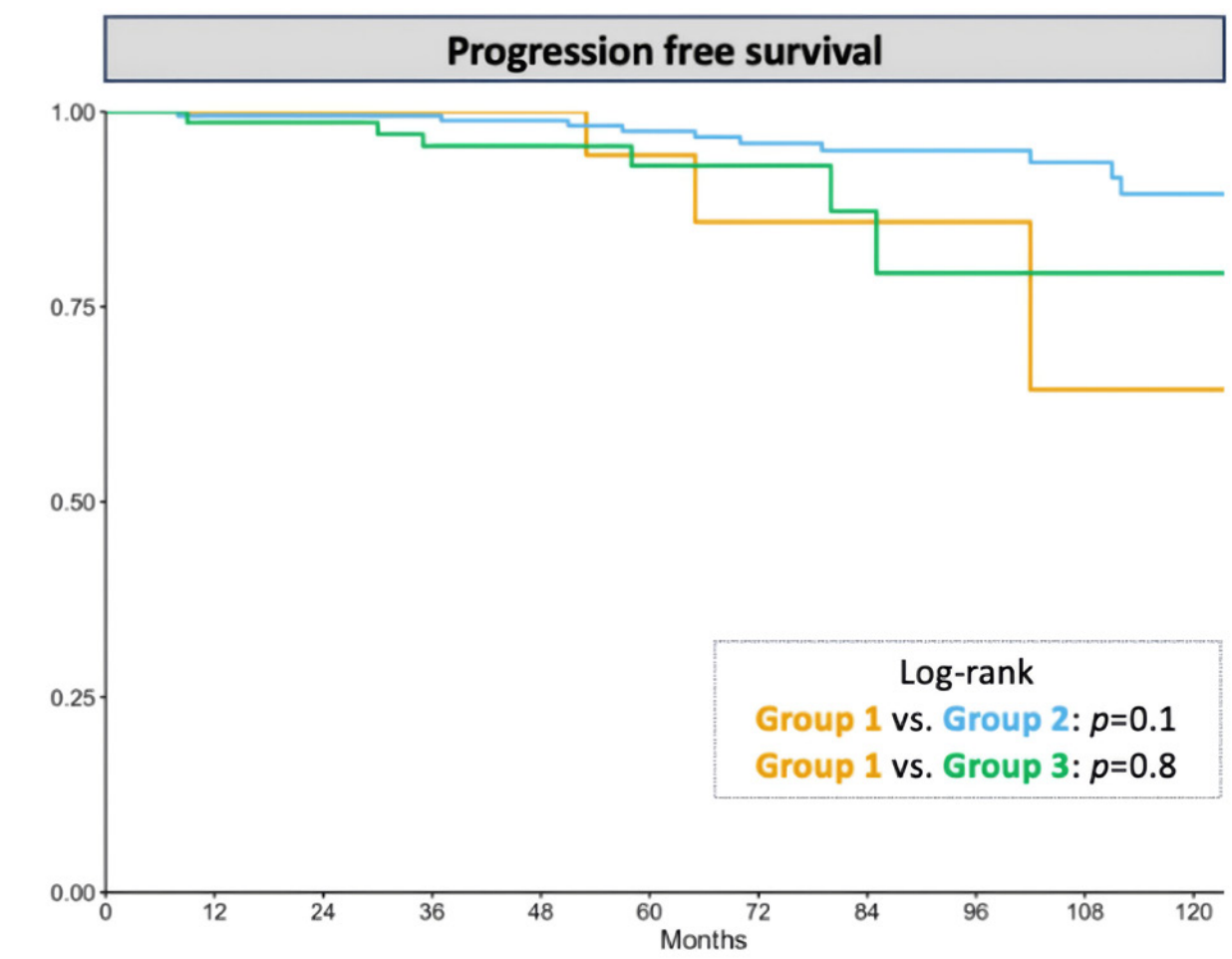

Months	0	12	24	36	48	60	72	84	96	108	120
Group 1	30	30	29	25	23	13	9	5	4	2	2
Group 2	181	179	174	167	152	135	116	98	72	52	34
Group 3	71	68	68	59	51	33	23	13	9	7	2

	1-year, % (95% CI)	2-years, % (95% CI)	3-years, % (95% CI)	5-years, % (95% CI)	10-years, % (95% CI)	Recurrence (months), Median (95% CI)
Group 1	100 (100-100)	100 (100-100)	100 (100-100)	94.4 (84.4-100)	64.4 (35.1-100)	NA (102-NA)
Group 2	99.4 (98.4-100)	99.4 (98.4-100)	99.4 (98.4-100)	97.5 (95.1-100)	89.5 (82.7-96.8)	NA (NA-NA)
Group 3	98.6 (95.8-100)	98.6 (95.8-100)	95.6 (90.8-100)	93.1 (86.5-100)	79.3 (62.6-100)	NA (NA-NA)

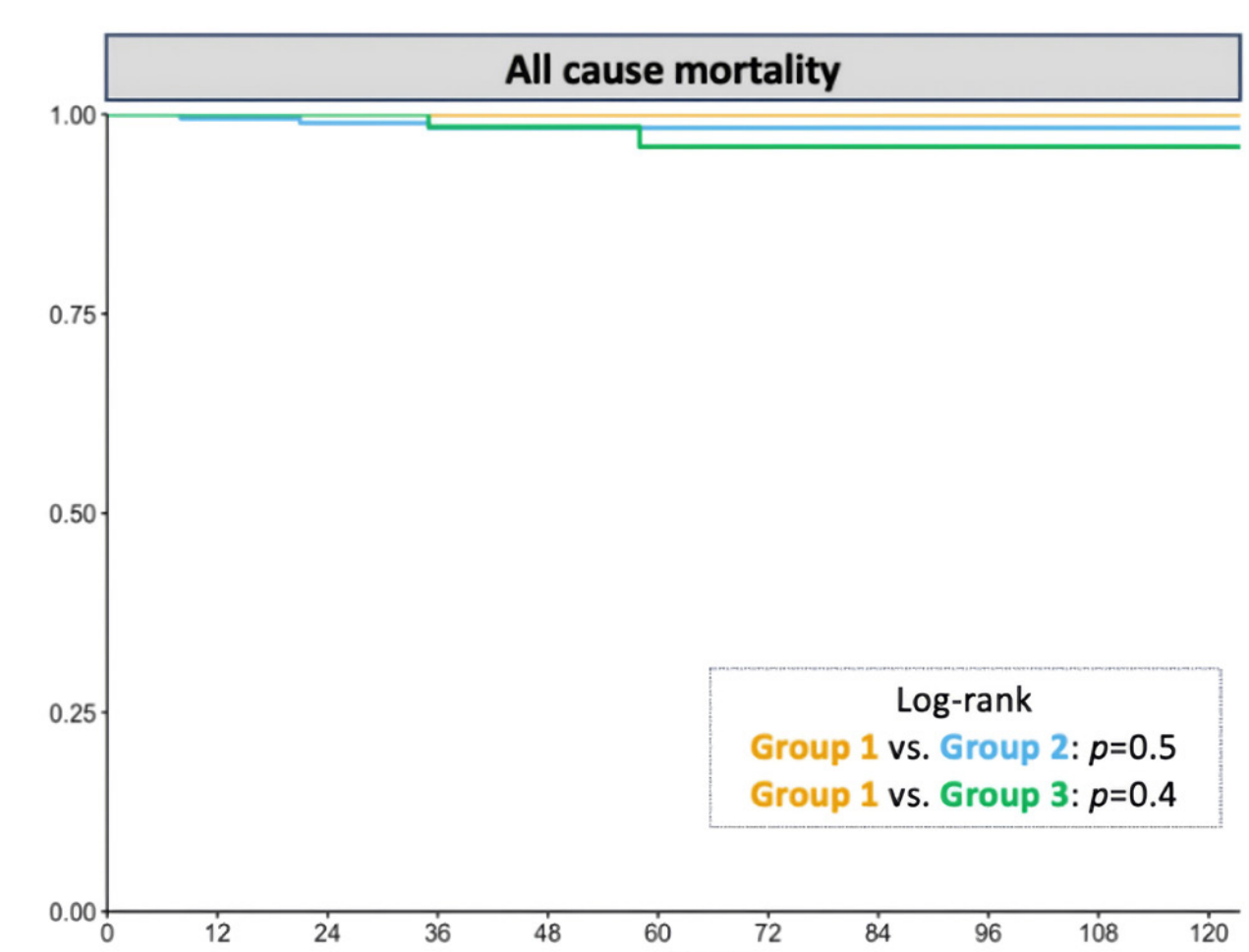

Months	0	12	24	36	48	60	72	84	96	108	120
Group 1	30	30	29	25	23	14	10	6	4	2	2
Group 2	181	180	175	168	155	140	122	104	76	55	38
Group 3	71	69	69	61	52	35	25	15	9	7	2

	1-year, % (95% CI)	2-years, % (95% CI)	3-years, % (95% CI)	5-years, % (95% CI)	10-years, % (95% CI)	Recurrence (months), Median (95% CI)
Group 1	100 (100-100)	100 (100-100)	100 (100-100)	100 (100-100)	100 (100-100)	NA (NA-NA)
Group 2	99.4 (98.4-100)	98.9 (97.4-100)	98.3 (96.4-100)	98.3 (96.4-100)	98.3 (96.4-100)	NA (NA-NA)
Group 3	100 (100-100)	100 (100-100)	98.4 (95.4-100)	95.9 (90.4-100)	95.9 (90.4-100)	NA (NA-NA)
